# Cytotoxicity Study of Cyclopentapeptide Analogues of Marine Natural Product Galaxamide towards Human Breast Cancer Cells

**DOI:** 10.1155/2017/8392035

**Published:** 2017-12-19

**Authors:** Jignesh Lunagariya, Xiaojian Liao, Weili Long, Shenghui Zhong, Poonam Bhadja, Hangbin Li, Bingxin Zhao, Shihai Xu

**Affiliations:** ^1^Department of Chemistry, College of Chemistry and Materials Science, Jinan University, 510632 Guangzhou, China; ^2^Institute of Biomineralization and Lithiasis Research, Jinan University, 510632 Guangzhou, China

## Abstract

Herein, we report the cytotoxicity of cyclopentapeptide analogues of marine natural product galaxamide towards breast carcinoma cells and the underlying mechanisms. We examined the effect of the novel galaxamide analogues on cancer cell proliferation by MTT assay and also further examined the most active compound for morphological changes using Hoechst33342 staining technique, induction of apoptosis, cell cycle phases, mitochondrial membrane potential (MMP), and reactive oxygen species (ROS) generation using flow cytometry in human breast cancer MCF-7 cells *in vitro*. Galaxamide and its analogues effectively induced toxicity in human hepatocellular carcinoma HepG2, human breast carcinoma MCF-7, human epitheloid cervix carcinoma HeLa, and human breast carcinoma MB-MDA-231 cell lines. Amongst them, compound 3 exhibited excellent toxicity towards MCF-7 cells. This galaxamide analogue significantly induced apoptosis in a dose-dependent manner in MCF-7 cells involves cell cycle arrest in the G1 phase, a reduction of MMP, and a marked increase in generation of ROS. Particularly, compound 3 of galaxamide analogues might be a potential candidate for the treatment of breast cancer.

## 1. Introduction

Over the past decade, substantial research has demonstrated that oxidative stress plays a crucial role in the development of most chronic diseases including different types of cancer [1]. All aspects of cancer, from carcinogenesis to the tumor-bearing state and from treatment to prevention, are closely associated with oxidative stress [2]. Some exogenous and endogenous processes drag the human body constantly under oxidative stress [3]. Oxidative stress generated in an excessive amount may introduce gene mutations or affect the intracellular signal transduction and transcription factors, which resulted in an increase of cell proliferation or a reduction of cell apoptosis, leading to carcinogenesis [4, 5]. Thus, the regulation of oxidative stress is an important factor in both tumor development and responses to anticancer therapies.

Cancer is a major public health problem worldwide and the second leading cause of death in the United States (US). Breast cancer is the second most common cause of cancer mortality among women worldwide [6]. In 2016, an estimated 246,660 new cases of breast cancer diagnosed among women and 2600 among men and approximately 40,450 women and 440 men are expected to die from breast cancer in the US [7]. Breast cancer incidence and death rates generally increase between the ages of 55 and 80. Fewer than 5 percent of breast cancers occur in women under the age of 40. The incidences of breast cancer are higher in developed countries than developing countries. Despite chemotherapeutic treatment of patients with metastatic breast cancer, resistance to therapy and cancer progression have been observed [8]. In this regard, much attention has been paid to active natural compounds for cancer treatment.

**Figure 1:**
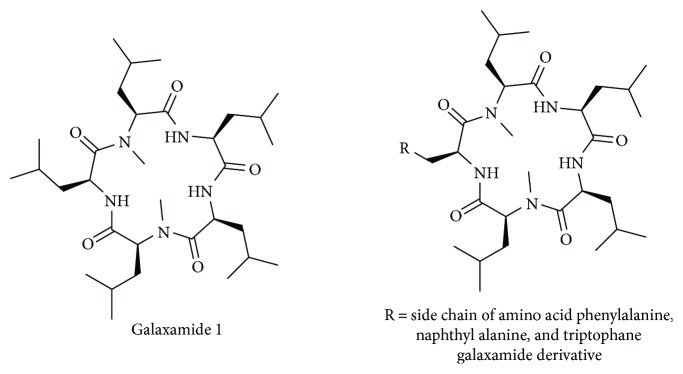
Structure of galaxamide and general structure of its derivatives.

In cancer therapy, marine natural products have occupied a broad area. A number of lead anticancer compounds from marine environment have grown rapidly in human clinical trials since the past decade. Recently, the antitumor activity of cyclic peptides has attracted much attention. A number of cyclopentapeptides have been synthesized and screened for their potential as anticancer agent. Firstly, Liu et al. [9] attempted *N*-methylated sansalvamide A analogue treatment for pancreatic cancer. Subsequently, Styers et al. have prepared many generation of sansalvamide A analogues for the treatment of colon and pancreatic cancer and found a number of potential lead compounds for the development of anticancer agent for pancreatic and colon cancer [10–12]. Liu et al. [13] discovered novel polypeptide Mere15 from *Meretrix meretrix* Linnaeus and found that the peptide was cytotoxic towards K562 leukemia cells via apoptosis induction, cell cycle arrest, and microtubule disassembly. Tran et al. [14] isolated novel cytotoxic peptides from the Australian marine sponge *Pipestela candelabra*, which inhibited growth of prostate cancer cells. Five new cyclic peptides, reniochalistatins A–E from marine sponge *Reniochalina stalagmitis*, were isolated and characterized by Zhan et al. [15] and screened for cytotoxic activity against various cancer cell lines. A recent study reported that a novel venom Rusvinoxidase peptide-induced intrinsic pathway of apoptosis in MCF-7 cells was accompanied by depolarization of the mitochondrial membrane through the generation of reactive oxygen species [16]. Recently, our group isolated and discovered galaxamide, an *N*-Methylated cyclic pentapeptide (Figure 1), from *Galaxaura filamentosa*. Its structural identification and total synthesis are also reported by our group [17]. Afterwards, several galaxamide analogues have been synthesized in our laboratory; amongst them, some analogues are found to be more active than galaxamide towards various cancer cell lines, such as HepG2 [18] and SMMC-7721 [19].

Apoptosis, a process of programmed cell death, is considered a vital mechanism to abolish cancer cells without eliciting damage to surrounding normal cells [20]. The understanding of apoptotic signaling pathways and insights into apoptosis resistance mechanisms have provided novel targets in the management and therapy for cancer that induce cell death to design and unravel cytotoxic drug compounds. Cell apoptosis involves two major death pathways, namely, the death receptor-mediated extrinsic pathway and the mitochondria-mediated intrinsic pathway [21]. The mitochondrial pathway of cell death can be activated by excessive generation of ROS [22]. It was documented that the production of ROS may cause changes in the inner mitochondrial membrane permeability, release of proapoptotic soluble protein, and triggering of caspase activation and also resulted in the loss of the mitochondrial membrane potential [23]. The activation of caspase family proteases permitted apoptosis progression [24]. Therefore, this pathway is an attractive target for anticancer therapy.

Herein, we designed and synthesized few analogues of naturally active anticancer agent galaxamide using solid-phase synthesis with replacing one l leucine amino acid residue with l phenyl alanine, l naphthyl alanine, and l triptophane (Figure 1). The novel analogues have been screened for their anticancer activity towards several carcinoma cell lines, such as HepG2, MCF-7, HeLa, and MB-MDA-231 using MTT assay. The most active compound 3 was further investigated to study the mechanism of cytotoxicity in human breast cancer MCF-7 cells.

## 2. Experimental Methods

### 2.1. Chemistry

#### 2.1.1. General

NMR spectra were recorded on a Bruker Advance 300 spectrometer (300 MHz for ^1^H and 75 MHz for ^13^C) in CDCl_3_. Chemical shifts are reported as *δ* values in parts per million (ppm) relative to tetramethylsilane (TMS) and *J* values are expressed in Hertz. The ESI mass spectra were obtained on a LCQ DECA XP LC-MS mass spectrometer. Silica gel (200–300 mesh) for column chromatography and silica GF254 for TLC was produced by Qingdao Marine Chemical Company (Qingdao, China). All air- or moisture-sensitive reactions were conducted under nitrogen atmosphere. Starting materials and reagents used in reactions were obtained commercially from Acros, Aldrich, GL Biochem, and were used without purification, unless otherwise indicated.

#### 2.1.2. Synthetic Procedure

Procedures for the synthesis of linear pentapeptide and subsequent macrocyclisation reaction have been followed as previously described in our article [19].

#### 2.1.3. Spectral Data

See supporting information for ^1^H, ^13^C, and ESI mass (positive and negative) spectra of compounds 1, 2, and 3.

L *Fmoc-N-Me Leucine*. Yield: 88%; ^1^H NMR (300 MHz, chloroform-*d*) *δ* ppm: 12.74 (s, 1H), 7.85 (t, *J* = 7.4 Hz, 2H), 7.60 (td, *J* = 7.6, 3.6 Hz, 2H), 7.43–7.23 (m, 4H), 4.64–4.17 (m, 4H), 2.68 (s, 3H), 1.74–1.19 (m, 3H), 0.89–0.63 (m, 6H); ^13^C NMR (75 MHz, chloroform-d) *δ* ppm:178.31, 156.24, 143.70, 141.35, 127.76, 127.11, 125.10, 120.03, 67.12, 52.42, 47.21, 41.44, 24.81, 22.91, 21.73; MS (ESI) *m*/*z*: 368.4 [M + H]^+^, 385.5 [M + NH_4_]^+^, 390.6 [M + Na]^+^.

*(1) Compound 1*. *cyclo(Phe-N-Me-Leu-Leu-Leu-N-Me-Leu)*. Yield: 60.9%, Wt: 59.3 mg, white powder; ^1^H NMR (300 MHz, chloroform-*d*) *δ* 0.84 (d, *J* = 6.3 Hz, 3H), 0.95 (dt, *J* = 17.8, 6.2 Hz, 22H), 1.33 (ddd, *J* = 13.5, 9.3, 4.6 Hz, 1H), 1.43–1.74 (m, 5H), 1.75–2.09 (m, 5H), 2.23–2.39 (m, 1H), 3.01 (t, *J* = 9.6 Hz, 8H), 3.35 (dd, *J* = 10.5, 4.6 Hz, 1H), 3.48 (t, *J* = 8.0 Hz, 1H), 4.25 (ddd, *J* = 10.9, 7.0, 3.9 Hz, 1H), 4.87 (q, *J* = 7.4 Hz, 1H), 5.09 (td, *J* = 9.6, 6.3 Hz, 1H), 5.86 (d, *J* = 7.1 Hz, 1H), 7.27 (q, *J* = 7.5 Hz, 7H), 8.46 (d, *J* = 9.5 Hz, 1H); ^13^C NMR (75 MHz, chloroform-*d*) *δ* 21.21, 21.48, 22.05, 22.67, 22.83, 23.31, 23.80, 24.50, 24.68, 25.28, 25.82, 127.04, 128.58, 129.18, 136.24, 170.27, 171.03, 172.11, 172.71, 173.49; MS (ESI) *m*/*z*: 628.7 [M + H]^+^, 645.7 [M + NH_4_]^+^, 650.7 [M + Na]^+^.

*(2) Compound 2*. *cyclo(Nal-N-Me-Leu-Leu-Leu-N-Me-Leu)*. Yield: 58.5%, Wt: 56.9 mg, white powder; ^1^H NMR (300 MHz, chloroform-*d*) *δ* 0.66 (dd, *J* = 16.9, 6.4 Hz, 6H), 0.87–1.02 (m, 18H), 1.23 (t, *J* = 7.0 Hz, 1H), 1.33 (ddd, *J* = 13.8, 9.1, 5.7 Hz, 1H), 1.46–1.61 (m, 4H), 1.77–1.92 (m, 6H), 2.02 (dt, *J* = 14.5, 7.7 Hz, 1H), 2.33 (dt, *J* = 14.4, 7.5 Hz, 1H), 3.03 (d, *J* = 7.6 Hz, 6H), 3.09–3.22 (m, 2H), 3.29 (dd, *J* = 10.0, 5.1 Hz, 1H), 3.50 (t, *J* = 8.2 Hz, 2H), 4.89 (q, *J* = 7.4, 6.9 Hz, 1H), 5.23 (td, *J* = 9.6, 6.1 Hz, 1H), 5.88 (d, *J* = 7.1 Hz, 1H), 7.40 (d, *J* = 8.4 Hz, 1H), 7.44–7.54 (m, 2H), 7.68 (s, 1H), 7.81 (dt, *J* = 15.7, 5.4 Hz, 3H); ^13^C NMR (75 MHz, chloroform-*d*) *δ* 21.19, 21.48, 22.02, 22.67, 22.85, 23.30, 24.52, 24.71, 25.29, 25.84, 37.19, 38.69, 38.85, 39.59, 40.63, 41.02, 41.24, 48.95, 50.13, 53.27, 125.86, 126.28, 127.12, 127.63 (d, *J* = 3.5 Hz), 127.87, 128.28, 170.26, 171.03, 172.22, 172.75; MS (ESI) *m*/*z*: 678.8 [M + H]^+^, 695.8 [M + NH_4_]^+^, 700.8 [M + Na]^+^.

*(3) Compound 3*. *cyclo(Trp-N-Me-Leu-Leu-Leu-N-Me-Leu)*. Yield: 55.5%, Wt: 54 mg, white powder ^1^H NMR (300 MHz, chloroform-*d*) *δ* 0.81 (dd, *J* = 15.9, 6.2 Hz, 6H), 0.95 (dt, *J* = 19.5, 5.8 Hz, 18H), 1.30 (dt, *J* = 14.1, 4.7 Hz, 1H), 1.54 (td, *J* = 12.4, 11.8, 6.7 Hz, 5H), 1.79–2.06 (m, 6H), 3.00 (d, *J* = 8.3 Hz, 6H), 3.14 (d, *J* = 7.9 Hz, 2H), 3.31 (dd, *J* = 10.5, 4.8 Hz, 1H), 3.50 (t, *J* = 8.2 Hz, 1H), 4.19–4.33 (m, 1H), 4.88 (q, *J* = 7.4 Hz, 1H), 5.26 (q, *J* = 8.4 Hz, 1H), 5.86 (d, *J* = 7.3 Hz, 1H), 7.07 (s, 1H), 7.12–7.27 (m, 2H), 7.37 (t, *J* = 8.9 Hz, 2H), 8.49 (d, *J* = 9.5 Hz, 1H); ^13^C NMR (75 MHz, chloroform-*d*) *δ* 21.20, 21.57, 22.00, 22.63, 22.74, 22.87, 23.31, 23.58, 24.55, 24.72, 25.28, 25.84, 28.36, 110.94 (d, *J* = 32.8 Hz), 118.74, 119.88, 122.45 (d, *J* = 5.5 Hz), 170.33, 171.11, 172.30, 172.66; MS (ESI) *m*/*z*: 667.7 [M + H]^+^, 684.9 [M + NH_4_]^+^, 689.7 [M + Na]^+^.

### 2.2. *In Vitro* Anticancer Activity

#### 2.2.1. Maintenance of Cell Culture

Human hepatocellular carcinoma HepG2, human breast carcinoma MCF-7, human epitheloid cervix carcinoma HeLa, human breast carcinoma MB-MDA-231 cell lines, and a normal human umbilical vein endothelial cell line (HUVECs) were obtained from the China Cell Bank of the Institute of Biochemistry and were cultured in a DMEM culture medium (DMEM, Corning, NY, USA) containing 10% fetal bovine serum (FBS, Hyclone, Logan, UT, USA), 1% penicillin-streptomycin, and an antifungal agent in a 5% CO_2_-humidified atmosphere at 37°C. The culture medium was replaced once in a day. Trypsin digestion method was adopted for cell propagation. Upon reaching 80–90% confluence, the cells were rinsed twice with PBS. A certain amount of 0.25% trypsin digestion solution was then added and maintained for 3–5 min at 37°C. Afterward, DMEM culture medium containing 10% fetal bovine serum was added to terminate the digestion. The cells were then blown well to form single-cell suspensions.

#### 2.2.2. MTT Assay

The cell viability was determined by measuring the ability of cells to transform MTT (3-(4,5-dimethylthiazol-2-yl)-2,5-diphenyltetrazolium bromide) (GENView, Houston, TX, USA) to a purple formazan dye [25]. The cells were seeded in 96-well cell culture plates at 2.5 × 10^3^ cells/well for 24 h. The cells were then incubated with galaxamide and its analogues for 48 h. After incubation, 20 *μ*L/well of MTT solution (5 mg/mL phosphate buffered saline) was added and incubated for 5 h. The medium was aspirated and replaced with 100 *μ*L/well of DMSO to dissolve the formazan salt. The color intensity of the formazan solution, which reflects the cell growth condition, was measured at 570 nm using a microplate spectrophotometer (SpectroAma™ 250, Winooski, VT, USA).

#### 2.2.3. Apoptotic Cell Analysis

The apoptotic cells were quantified using an Annexin V-FITC (fluorescein isothiocyanate) (Sigma-Aldrich, St. Louis, MO, USA) cell apoptosis assay kit according to the instruction provided with the kit [25]. In brief, about 1.5 × 10^5^ cells were plated in 6-well plates and treated with galaxamide and representative compound (0, 2.5, 5, and 10 *μ*g/mL) for 48 h. The cells were resuspended in 200 mL binding buffer. Afterwards, 5 mL Annexin V-FITC was added and then incubated in darkness at room temperature for 10 min. The cells were again resuspended in 200 mL binding buffer and stained with 5 mL PI (propidium iodide). The prepared cells were then analyzed using a flow cytometry (Coulter Epics Elite, Miami, FL, USA). The cells in the FITC-positive and PI-negative fractions were regarded as apoptotic cells.

#### 2.2.4. Cell Morphological Observation

Exponentially growing MCF-7 cells (1 × 10^5^ cells/mL) were incubated for 48 h with 0, 2.5, 5, and 10 *μ*g/mL of galaxamide and representative analogue. Apoptotic nuclear morphology was visualized by the Hoechst33342 staining technique. Cells were fixed with 3.7% of paraformaldehyde for 10 min at room temperature and washed three times with PBS. After fixation, cells were stained using Hoechst33342 (10 *μ*g/mL) and incubated in the darkness for 10 min. After washing three times with PBS, cells were visualized using fluorescence microscope (IX51, Olympus, Japan).

#### 2.2.5. Cell Cycle Analysis

MCF-7 cells with the density of about 1.5 × 10^5^ were incubated with galaxamide and representative analogue (0, 2.5, 5, and 10 *μ*g/mL) for 48 h. Afterwards, collected cells were washed twice with PBS and centrifugation (1000 rpm, 5 min), then fixed using 70% ethanol for 12 h at 4°C. Ethanol was removed by centrifugation (2000 rpm, 5 min), and the cells were washed twice with PBS. Cells were then resuspended in 200 mL PI and kept at 37°C for 15 min. The cell cycle was analyzed by flow cytometry measuring the amount of PI-labeled DNA in fixed cells.

#### 2.2.6. Measurement of Mitochondrial Membrane Potential

The cell suspension (2 mL) with a concentration of 1 × 10^5^ cells/mL was inoculated per well in 6-well plates for 12 h. After 48 h of incubation with galaxamide and representative compound at the concentration of 0, 2.5, 5, and 10 *μ*g/mL, the supernatant was aspirated and the cells were washed twice with PBS and digested with 0.25% trypsin. The cells were suspended by pipetting, followed by centrifugation (1000 rpm, 5 min). The supernatant was aspirated and the cells were washed with PBS and centrifuged again to obtain a cell pellet. The cells were resuspended by adding and thoroughly mixing 500 *μ*L of PBS in a microcentrifuge tube. Finally, the samples were stained with JC-1 (5,5′,6,6′-tetrachloro-1,1′,3,3′-tetraethylbenzimi-dazolylcarbocyanine iodide) and incubated in darkness at 37°C for 15 min. After the treatment, the cells were detected by flow cytometry.

#### 2.2.7. Intracellular ROS assay

Two milliliters of cell suspension with a cell concentration of 1 × 10^5^ cells/mL was inoculated per well in 6-well plates. After synchronization, the cells were incubated with galaxamide and representative analogue (0, 2.5, 5, and 10 *μ*g/mL) for 48 h. Afterwards, the supernatant was aspirated and the cells were washed twice with PBS and digested with 0.25% trypsin. Then, DMEM supplemented with 10% fetal bovine serum was added to terminate digestion. The cells were suspended by pipetting, followed by centrifugation (1000 rpm, 5 min). The supernatant was aspirated and the cells were washed once with PBS and centrifuged again to obtain a cell pellet. The cells were resuspended by adding and thoroughly mixing 500 mL PBS in a microcentrifuge tube. The samples were then stained with DCFH-DA (20,70-dichloro-fluorescein diacetate) and analyzed by flow cytometry.

#### 2.2.8. Statistical Analysis

Experimental data were expressed as mean ± standard deviation (SD). The experimental results were analyzed statistically using SPSS software. The statistical analysis were performed using the Student's *t*-test; differences between concentrations were considered significant when *p* < 0.05.

## 3. Results

### 3.1. Chemistry

The solid-phase synthesis method has been developed for the linear pentapeptide using 2-choloro trityl resin. Previously, it has been tried by 2 + 3 fragment-based solution phase strategy by many scientists for the synthesis of cyclopentapeptide [10–12, 17–19]. The preparation of L*N*-Me Leucine has been carried out according to the reported method by Zhang et al. [26]. Initially, amino acid residue was immobilized on resin using 4.0 eq. *N*,*N*-diisopropylethylamine (DIPEA) in dichloromethane (DCM) for 2 h at room temperature (rt). Subsequently, amino acid residue including *N*-Me amino acid was coupled with 3-(diethoxyphosphoryloxy)-1,2,3-benzotriazin-4(3*H*)-one (DEPBT) activation using DIPEA as base in DCM for 3 h at rt. Solid-phase reactions were monitored by means of a qualitative Kaiser test [27] for the detection of primary amines and the chloranil test [28] for detection of secondary amines. Upon cleavage from solid support with 1% trifluoroacetic acid (TFA) in DCM, linear peptide was obtained in 55–60% overall yield and 95–99% purity which was measured by RP-HPLC.

**Figure 2:**
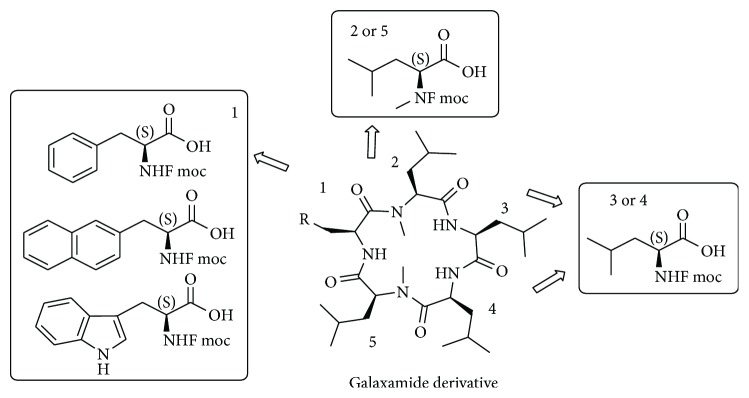
Amino acid used at various positions for galaxamide derivatives.

**Figure 3:**
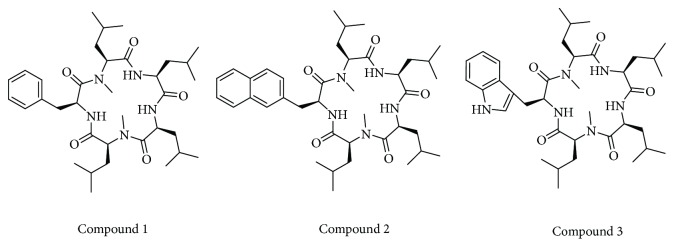
Structure of the synthesized cyclopentapeptide compounds.

The isolation of linear pentapeptide was carried out by precipitation with diethyl ether, which was collected by centrifuge and can be used without further treatment for the next macrocyclisation reaction. Macrocyclisation reaction was performed using benzotriazol-1-yloxy tripyrrolidinophosphonium hexafluorophosphate (PyBOP) with 0.0007 M dilution of solvent DCM and 2 eq. of DIPEA. After 24 h reaction at rt, desired macrocycles were obtained in 45–60% yield through RP-HPLC purification.  Figure 2 shows the amino acid employed in the sequence of linear peptide as schematic diagram. The structure of the synthesized final compound of the cyclopentapeptide is displayed in Figure 3.

### 3.2. Anticancer Activity

#### 3.2.1. Galaxamide Analogues Exhibited Cytotoxicity towards Cancer Cells

In this study, galaxamide and its three analogues were evaluated for their cytotoxic effect against the panel of human hepatocellular carcinoma HepG2, human breast carcinoma MCF-7, human epitheloid cervix carcinoma HeLa, human breast carcinoma MB-MDA-231 cell lines, and a normal human umbilical vein endothelial cell line (HUVECs) using MTT assay. As shown in Table 1, compound 1 exhibited less cytotoxicity after 48 h against HepG2 and HeLa cells compared to galaxamide and other derivatives, with IC_50_ values of 6.25 *μ*g/mL and 13.22 *μ*g/mL, respectively. The IC_50_ values of compound 2 were 8.42 *μ*g/mL in HepG2, 3.16 *μ*g/mL in MCF-7, 13.22 *μ*g/mL in HeLa, and 4.48 *μ*g/mL in MDA-MB-231 cells (Table 1), displaying moderate activity. Whereas, cytotoxicity study noticeably demonstrated that the compound 3 is relatively potent against all the cancer cell lines as compared to galaxamide (*p* < 0.05) and other derivatives; the IC_50_ values were determined to be 3.98 *μ*g/mL towards HepG2 (versus compounds 1 and 2, *p* < 0.05), 1.72 *μ*g/mL towards MCF-7 (versus compound 1, *p* < 0.05), 5.32 *μ*g/mL towards HeLa (versus compound 1 and 2, *p* < 0.05), and 3.51 *μ*g/mL towards MD-MBA-231 (versus compounds 1, *p* < 0.05) cells after 48 h (Table 1). All the compounds presented higher cytotoxicity against cancer cells than approved anticancer drug cisplatin but lower than doxorubicin. Moreover, present investigation indicated that the analogues of galaxamide exhibited greater toxicity towards MCF-7 cells compared to other cancer cells. However, significant difference was not found between MCF-7 and MDA-MB-231 cells in case of compounds 1 and 2, as both the cell lines are from human breast cancer. Hence, we selected compound 3 for further study of the mechanism of cytotoxicity against MCF-7 cells. Additionally, galaxamide and its analogues demonstrated lower toxicity on normal (HUVECs) cells (IC_50_ > 40 *μ*g/mL).

#### 3.2.2. Galaxamide Analogue-Induced Cell Apoptosis

**Figure 4:**
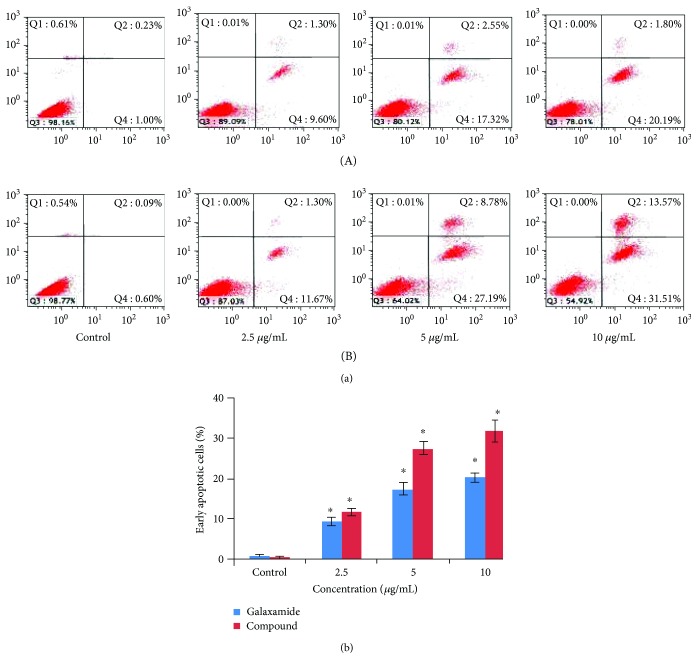
Galaxamide and its analogue induced apoptosis in human breast cancer MCF-7 cells. (a) MCF-7 cells were treated with 0, 2.5, 5.0, and 10.0 *μ*g/mL galaxamide (A) and compound 3 (B) for 48 h. The apoptosis of MCF-7 cells was determined by Annexin V/PI staining. Q1 (the upper left) quadrant represents necrotic cells, Q2 (the upper right) quadrant represents late apoptotic and dead cells, Q3 (the bottom left) represents normal cells, and Q4 (the bottom right) quadrant represent early apoptotic cells. (b) Annexin V-positive cells of three independent experiments were shown in column statistics. Data expressed as mean ± SD. ^∗^*p* < 0.005 versus control.

Cell apoptosis was assessed by Annexin V-FITC/PI staining in MCF-7 cells treated with galaxamide and its analogue for 48 h at different concentrations (2.5, 5 and 10 *μ*g/mL). Compound 3 significantly induced the apoptosis of human breast cancer MCF-7 cells in a dose-dependent manner (Figure 4). After 48 h of 10 *μ*g/mL compound 3 treatment, most MCF-7 cells were undergoing apoptosis.

**Figure 5:**
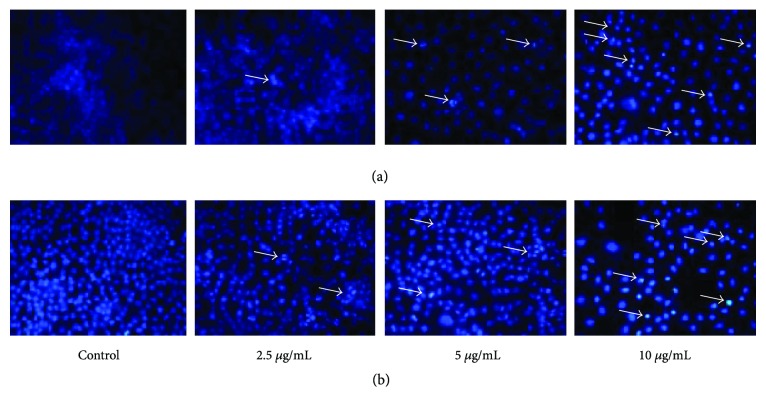
Nuclear morphology of MCF-7 cells treated with 0, 2.5, 5.0, and 10.0 *μ*g/mL galaxamide (a) and compound 3 (b) for 48 h was determined by staining with Hoechst 33258. Arrows indicate apoptotic cells.

To further determine galaxamide analogue-induced apoptosis in MCF-7 cells, we analyzed morphological nuclear changes using Hoechst33258 staining. As shown in Figure 5, treatment of MCF-7 cells with galaxamide and its analogue induced membrane blebbing, condensation and disintegration of chromatin, DNA fragmentation, and shrinkage of cells. These morphological features showed that the compound shares cell growth inhibition by inducing apoptosis of cancerous cells.

#### 3.2.3. Galaxamide Analogue-Induced Cell Cycle Arrest in the G1 Phase

**Figure 6:**
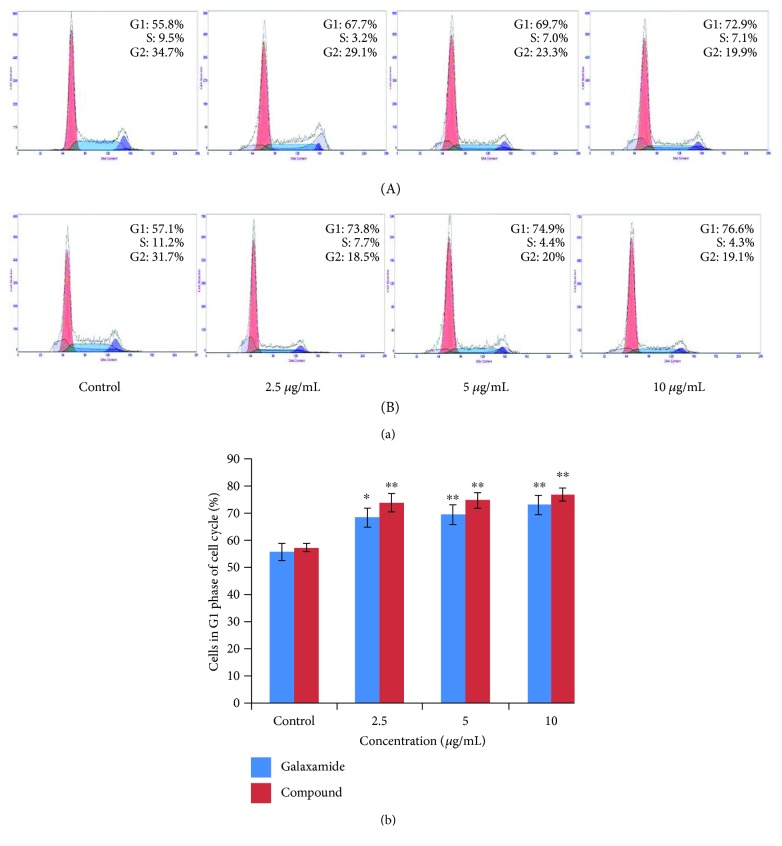
Galaxamide and its analogue triggered cell cycle arrest in MCF-7 cells. (a) Galaxamide (A) and compound 3 (B) at 0, 2.5, 5.0, and 10.0 *μ*g/mL caused G1 phase cell cycle arrest after 48 h were detected by PI staining using flow cytometry. (b) Data are mean ± SD of three independent experiments. ^∗^*p* < 0.05, ^∗∗^*p* < 0.01 versus control.

Cell cycle arrest is one of the major causes of cell death. To investigate whether galaxamide analogue-induced apoptosis was associated with cell cycle arrest, we studied cell cycle distribution in MCF-7 cells using flow cytometry to analyze the DNA content in each cell cycle phase. Results of this experiment showed that treatment of galaxamide and its derivative promoted a dose-dependent increase in the amount of cells in the G1 phase compared to the untreated control (Figure 6). The data suggested that compound 3 caused MCF-7 cell death through cell cycle arrest in the G1 phase and by the induction of apoptosis.

#### 3.2.4. Galaxamide Analogue-Induced Mitochondria-Mediated Apoptosis

**Figure 7:**
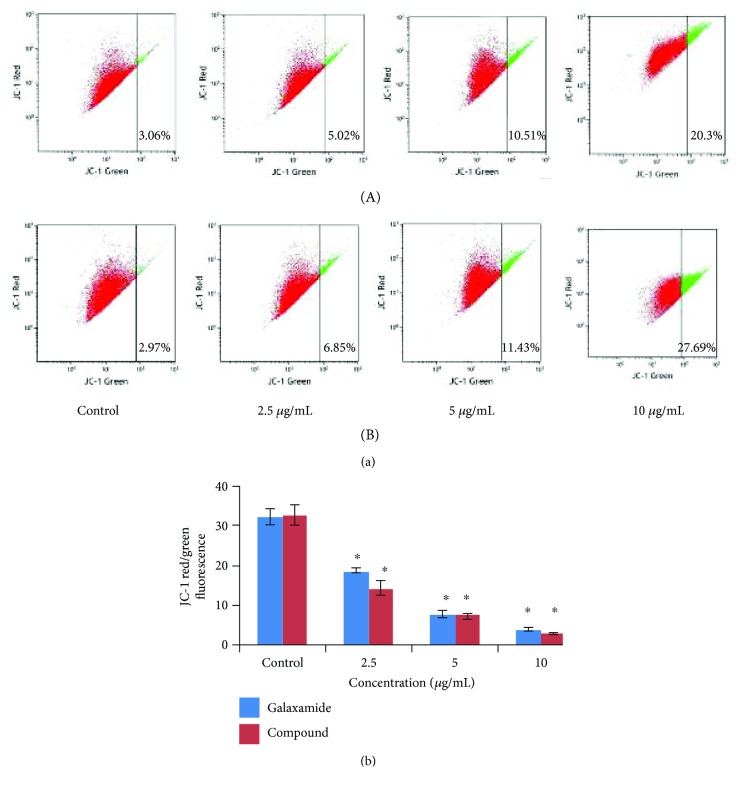
The effect of galaxamide and its analogue on the mitochondrial membrane potential (MMP) in MCF-7 cells. (a) Cells incubated with galaxamide (A) and compound 3 (B) at 0, 2.5, 5.0, and 10.0 *μ*g/mL for 48 h were stained with JC-1, the ratio of JC-1 red/green fluorescence was determined by flow cytometry. (b) The loss of the MMP in MCF-7 cells in a dose-dependent manner. Data are mean ± SD of three independent experiments. ^∗^*p* < 0.005 versus control.

Mitochondrial stress pathway is one of the most significant intracellular signaling followed in apoptosis. To investigate whether galaxamide analogue-induced cell apoptosis was associated with mitochondrial dysfunction, we analyzed changes in mitochondrial membrane potential (MMP) using JC-1 staining, a mitochondria sensitive dye, and examining the cells by flow cytometry. Results demonstrated that the fluorescence ratio (red/green) in MCF-7 cells was reduced dose dependently by galaxamide and compound 3 treatment (Figure 7), indicating that compound 3 disrupted MMP and eventually induced cell apoptosis through the intrinsic pathway.

#### 3.2.5. ROS Involved in the Galaxamide Analogue-Induced Apoptosis

**Figure 8:**
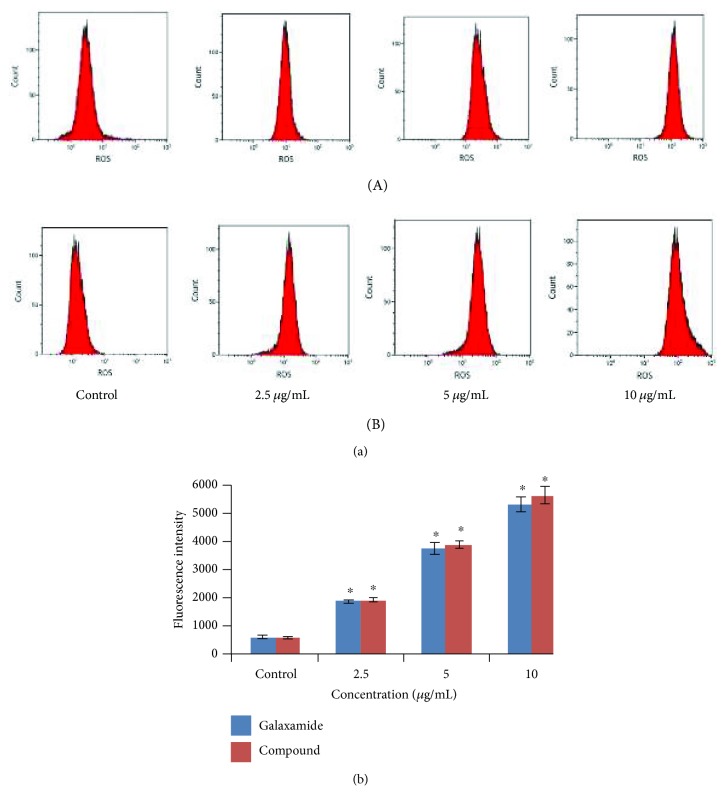
Galaxamide and its analogue induced ROS production in breast cancer cells. (a) MCF-7 cells were treated with different concentrations (0, 2.5, 5.0, and 10.0 *μ*g/mL) of galaxamide (A) and compound 3 (B) for 48 h, and then ROS was measured by DCFH-DA fluorescence analysis using flow cytometry. (b) The increased fluorescence of DCFH-DA was determined as the increased intracellular ROS accumulation. Data expressed as mean ± SD of three independent experiments. ^∗^*p* < 0.005 versus control.

As ROS production is an important factor in mitochondrial-induced apoptosis, the effect of galaxamide and its derivative on the generation of ROS was investigated in MCF-7 cells by DCFH-DA staining using flow cytometry. As illustrated in Figure 8, treatment with galaxamide and its analogue for 48 h significantly increased intracellular ROS in dose-dependent manner in MCF-7 cells. This result suggests that compound 3 may induce cell apoptosis by the generation of ROS in breast cancer cells.

## 4. Discussion

With the advent of drug discovery from marine natural products, it has been continued to be a powerful approach for the search of biologically active compounds for the identification of novel anticancer chemotherapeutic agents in order to develop new therapies in cancer [29]. As a part of our ongoing project on the discovery of novel biologically active compound from marine natural product and synthesis of their analogues, the aim of this investigation was to elucidate the mechanism of antitumor activity of galaxamide analogue-a cyclopeptide, on human breast cancer cells, which may provide a potential alternate for the drug discovery and treatment of breast cancer.

Radiation and chemotherapy are the major approaches to treat human breast cancer [30]. However, the conventional chemotherapy destroys rapidly dividing cells, which can also affect the normal dividing cells; similarly, radiation therapy used to damage cancer cells, also damage healthy cells [31]. In addition, with the use of anticancer drugs, severe toxicities and drug resistance often occur [32]. Consequently, considerable interest has been focused on natural bioactive compounds and their derivatives capable of inhibiting the growth of breast cancer. Along the same line, we have been attracted in discovering novel, effective, and safe drugs from marine natural products for cancer therapy. Galaxamide is a cyclic pentapeptide isolated and identified from marine algae *Galaxaura filamentosa* with the first total synthesis by our group [17]. Subsequently, Xiao et al. [18] and Lunagariya et al. [19] developed several analogues with modifications that are projected towards increasing potency and effectiveness. In previous studies, comparison of the analogues with the parent compound revealed an increased potency in some of the analogues towards HepG2 [18] and SMMC-7721 [19] carcinoma cell lines. In the present investigation, a total of three compounds were synthesized using the solid-phase synthetic method by replacing one amino acid residue with L-phenyl alanine, L-naphthyl alanine, and L-triptophane (Figure 3). There are several reports that have been published on sansalvamide A derivative which have shown potent cytotoxicity towards various cancer cell line [9–12]. This structure contains phenyl alanine residue and the rest is almost similar to galaxamide. Few reports on sansalvamide A suggested that the every position has their effect on cytotoxicity individually. However, when we combined all changes at a specific position, it would not provide synergistic effect [33]. Hence, upon basis of that result herein, we have mainly focused on hydrophobic aromatic moiety at a particular position in galaxamide and observed the alteration in cytotoxicity with the changes in aromatic structure including hydrogen-bonding element (compound 3). Galaxamide analogues demonstrated a particular trend in toxicity across all cell lines, such as compound 1 with phenyl ring showing lower activity than other analogues, compound 2 displaying higher activity than compound 1 and lower than compound 3, and compound 3 exhibiting lower cytotoxicity than compound 1 and 2. Especially, compound 3-containing hydrogen bond element –NH showed almost sixfold higher cytotoxicity than parent structure galaxamide, at the same time which is 8.49-fold more active than anticancer drug DPP and 3.44-fold less active than current drug doxorubicin (Table 1). The more potent compound 3 of these analogues was employed for the further study which focused on depicting the mechanism of induction of apoptosis in human breast cancer cells (MCF-7). Moreover, it should be noted that the toxic effect of galaxamide analogues was tumor cell selective, as analogues did not affect the viability of nontumor cells. Thus, galaxamide and its analogues might be considered as valuable candidate for treatment of breast cancer compare to other adaptive therapies.

In the examination for the mechanism of this cyclopeptide against breast cancer, compound 3 found to induce apoptosis in MCF-7 cells (Figure 4). Apoptosis is an acquired hallmark of cancer cells and known to play a vital role in tissue homeostatic balance. Alterations or defiance against apoptosis mechanisms can lead to cancer pathogenesis [34]. Thus, improving cell apoptosis events and therapeutic efficacy are imperative in developing antitumor agents. Numerous anticancer agents inhibit tumors by targeting apoptotic pathways [35, 36]. Apoptosis is characterized by distinct cell morphological characteristics, such as nuclear condensation, DNA fragmentation, cell shrinkage, and cell membrane disintegration [19, 24]. Our morphological analysis also displayed similar types of apoptotic features (Figure 5).

The development of cancer has been revealed to involve the uncontrolled cell cycle checkpoints that regulate passage through the cell cycle [37]. When specific checkpoints during the cell cycle are arrested, the programmed cell death occurs [24, 38]. Importantly, the regulation of cell cycle progression and division in cancer cells is considered to be an effective event for the management of tumor growth [39, 40]. It has been stated that cyclic-peptide sansalvamide analogues induce apoptosis in pancreatic cancer cell through G0/G1 cell cycle arrest [38]. Herein, from the outcome of the flow cytometry study, we observed that the treatment of MCF-7 cells with compound 3 induced a significant G1 phase arrest of cell cycle progression in a dose-dependent manner (Figure 6), which suggested that one of the mechanisms by which galaxamide derivative may act to inhibit the proliferation of cancer cells is inhibition of cell cycle progression. The apoptotic pathway is to be followed via the G0/G1 phase arrest of cell cycle regulation. The several cyclin-dependent kinase (CDK) complexes controlled the G1 to S cell cycle progression, and it is dependent on the balance of cyclins and cyclin-dependent kinase inhibitors (CKIs) [41]. p53, the most extensively studied tumor suppressor, mediates a variety of antiproliferative processes through cell cycle checkpoints, DNA repair, and apoptosis [42]. Previous investigation has demonstrated that p21 is one of the most important CKIs to directly inhibit the activity of CDKs, consequently, leading to cell cycle arrest in the G1 phase [43]. Hence, this may slow down the development of cancer cells by artificially imposing the cell cycle checkpoint.

Mitochondrial stress pathway is the crucial step in apoptosis induction which could be witnessed via the depolarization of MMP. The involvement of the mitochondrial pathway in cell apoptosis was confirmed by alteration in MMP [44]. Cell apoptosis induced by the activation of several death effector caspases, which would be resulted from the releases of cytochrome c from the intermembrane space of mitochondrion to cytosol due to the depolarization of the mitochondrial membrane [45]. In present investigation, to detect the involvement of the mitochondrial pathway in apoptosis induction, JC-1 staining was used, which observed the changes in MMP. The data of JC-1 staining demonstrated that the MMP in MCF-7 cells was significantly reduced by treatment of galaxamide and compound 3 (Figure 7). This result revealed that compound 3 induced apoptosis in MCF-7 cells through the mitochondrial pathway. Our laboratory has published a report which demonstrated that the mitochondrial pathway was followed through activation of caspases for the induction of apoptosis [18]. It usually involved Bcl-2 family proteins, whose members may be antiapoptotic and proapoptotic proteins, control mitochondrial membrane permeability during apoptosis, and finally regulate cell death [24]. PARP cleavage, which resulted through the activation of the caspase cascade, is considered as a key factor in the apoptotic signaling pathway [45]. Bax, an antagonist of Bcl-2, is inserted into the outer membrane of the mitochondria, permitting for the release of cytochrome c and activating caspase-9 [46]. Subsequently, proteolytic cleavage of caspase-9 activated caspase-3, which is a main apoptotic executive caspase [47].

ROS generation has been shown to be involved as the imperative mediator and essential for inducing apoptosis for several types of cancers [48, 49]. Although mitochondria are believed to be a predominant site of ROS production, overexpression of ROS may lead to the free radical attack of membrane phospholipids and loss of MMP, which causes the release of apoptosis-inducing factors that activate caspase cascades and cause nuclear condensation [48]. Mitochondrion-mediated apoptosis is supposed to be occurring through ROS generation mechanism [50]. Moreover, a large amount of ROS can inhibit tumor growth through the sustained activation of cell cycle inhibitors [51]. Our results described that galaxamide and compound 3 induced ROS in a dose-dependent manner (Figure 8), which may participate in apoptosis as well.

## 5. Conclusion

Taken together, our results demonstrated that galaxamide analogue induced apoptosis, mediated by disrupting membrane potential of mitochondria, eliciting production of ROS, and activation of the oxidative stress-mediated signaling pathway in breast cancer cells. In addition, the generation of ROS led to cell cycle arrest, which is involved in galaxamide analogue-induced MCF-7 cell apoptosis. Further experimental work will confirm the precise targeting pathway of apoptosis. Our investigation thus provides a rational mechanism for the development of anticancer agents.

## Figures and Tables

**Table 1:** Cytotoxicity of galaxamide and its analogues towards various cancer cell lines and normal cells as determined by MTT assay. Data expressed as mean ± SD from three independent experiments.

Compound	IC_50_ (*μ*g/mL) values in various cell lines				
	HepG2	MCF-7	HeLa	MD-MBA-231	HUVECs
Galaxamide	4.23 ± 0.18	10.25 ± 1.89	9.75 ± 1.36	8.27 ± 0.94	>40
1	6.25 ± 1.03	4.76 ± 1.36	13.22 ± 1.12	5.83 ± 0.45	>40
2	8.42 ± 1.82	3.16 ± 0.92	6.43 ± 1.20	4.48 ± 2.24	>40
3	3.98 ± 0.71	1.72 ± 0.85	5.32 ± 0.42	3.51 ± 1.32	>40
DPP	10.43 ± 1.23	14.61 ± 2.01	8.90 ± 0.89	13.75 ± 0.56	
Doxorubicin	2.27 ± 0.24	0.50 ± 0.48	0.32 ± 0.22	1.29 ± 0.57	

## Supplementary Materials

Supplementary Materials Spectral data. S-2: ^1^H and ^13^C NMR spectra of compound 1. S-3: ESI Mass Spectrum (Positive and negative mode) of compound 1. S-4: ^1^H and ^13^C NMR spectra of compound 2. S-5: ESI Mass Spectrum (Positive and negative mode) of compound 2. S-6: ^1^H and ^13^C NMR spectra of compound 3. S-7: ESI Mass Spectrum (Positive and negative mode) of compound 3.
