# Supplementary material for: Cytotoxicity Study of Cyclopentapeptide Analogues of Marine Natural Product Galaxamide towards Human Breast Cancer Cells
Source: Oxid Med Cell Longev. 2017 Dec 19;2017:8392035. doi: 10.1155/2017/8392035 (PMC5749289; doi:10.1155/2017/8392035)
Supplement: Supplementary Materials — Spectral data. S-2: 1H and 13C NMR spectra of compound 1. S-3: ESI Mass Spectrum (Positive and negative mode) of compound 1. S-4: 1H and 13C NMR spectra of compound 2. S-5: ESI Mass Spectrum (Positive and negative mode) of compound 2. S-6: 1H and 13C NMR spectra of compound 3. S-7: ESI Mass Spectrum (Positive and negative mode) of compound 3. [file 8392035.f1.docx]

**Cytotoxicity Study of Cyclo-pentapeptide Analogues of Marine Natural Product Galaxamide towards Human Breast Cancer Cells**

Jignesh Lunagariya^1#^, Xiaojian Liao^1#^, Weili Long^1^, Shenghui Zhong^1^, Poonam Bhadja^2^, Hangbin Li^1^, Shihai Xu^1^*, Bingxin Zhao^1^*

^1^Department of Chemistry, Life Science School, Jinan University, Guangzhou 510632, China

^2^Institute of Biomineralization and Lithiasis Research, Jinan University, Guangzhou 510632, China

**Supporting Information**

**Spectral data**

^1^H and ^13^C NMR spectra of **compound 1** S-2

ESI Mass Spectrum (Positive and negative mode) of **compound 1** S-3

^1^H and ^13^C NMR spectra of **compound 2** S-4

ESI Mass Spectrum (Positive and negative mode) of **compound 2** S-5

^1^H and ^13^C NMR spectra of **compound 3** S-6

ESI Mass Spectrum (Positive and negative mode) of **compound 3** S-7

**Compound 1**

^1^H NMR

^13^C NMR

ESI Mass

Positive mode

Negative mode

**Compound 2**

^1^H NMR

^13^C NMR

ESI Mass

Positive mode

Negative mode

**Compound 3**

^1^H NMR

^13^C NMR

ESI Mass

Positive mode

Negative mode
